# Muscle architecture of the medial gastrocnemius during growth

**DOI:** 10.1186/s40101-024-00381-4

**Published:** 2024-12-30

**Authors:** Yasuyoshi Mogi

**Affiliations:** Faculty of Sport Management, Department of Sport Management, Shobi University, 1-1-1, Toyoda-cho, Kawagoe, Saitama 350-1110 Japan

**Keywords:** Muscle thickness, Pennation angle, Fascicle length, Children, Adolescents

## Abstract

**Background:**

Muscle architecture is closely related to muscle function. Increased knowledge of growth changes in muscle architecture will provide insights into the development of human movements and sports performance during the growth period. However, it is unclear how the muscle architecture of the medial gastrocnemius (MG) grows. This study examined the effects of growth on the muscle architecture of MG.

**Methods:**

The brightness-mode ultrasonography technique was used to measure the muscle thickness, pennation angle, and fascicle length of MG in 146 Japanese boys aged to 6.2 − 17.9 years. The relative muscle thickness was calculated by dividing the absolute muscle thickness by body mass^1/3^. The years from the age at peak height velocity were estimated for each participant, and used as the maturity index. A simple regression analysis was performed for the two variables in the full age range, as well as separately for the 5 − 12 years and 12 − 19 years subgroups.

**Results and conclusion:**

The maturity index and chronological age were positively correlated with the relative muscle thickness, pennation angle, and fascicle length of MG. Subgroup analyses showed that chronological age was significantly correlated with the pennation angle, fascicle length, and absolute muscle thickness, except for the pennation angle of the 5 − 12 years subgroup. The present results indicate that muscle hypertrophy and elongation of fascicle length occur with growth. Our findings also suggest that the growth changes in pennation angle of MG differ between pre-adolescence and adolescence.

## Background

Muscle architecture is related to muscle functions. For example, muscle thickness and pennation angle affect muscle force production [1, 2]. Fascicle length is closely related to muscle shortening velocity [3] and the force–length relation of muscle [1]. Therefore, it is quite important to investigate the effects of growth on muscle architecture to explore the development of human movement during growth.

During the growth period, there is a proportional relationship between chronological age and the pennation angle and the muscle thickness for the medial gastrocnemius (MG) [4]. Weide et al. [5] also showed that chronological age was positively and significantly correlated with pennation angle, but no such correlation was observed for fascicle length. In addition, Pentidis et al. [6] examined the growth changes in the muscle thickness, pennation angle, and fascicle length of MG in children. The results showed that the muscle thickness and pennation angle increased, but the fascicle length remained unchanged. These findings suggest that the muscle thickness and pennation angle increase with growth, but fascicle length does not change. On the other hand, only one study [7] reported that the muscle thickness and fascicle length of MG were correlated with chronological age and that no such correlation was observed for the pennation angle. These results are in contrast to those of previous studies [5–7], which observed an increase in pennation angle with growth but unchanged the fascicle length. The discrepancy between the previous findings may be attributed to the relatively small number of participants ([7]: *n* = 30, [6]: *n* = 32, [5]: *n* = 16) and the narrow age range ([7]: 5 − 12 years, [6]: from 9 to 10 years, [5]: 10 − 19 years). Therefore, further research with a larger sample size and a wider age range is needed to gain a comprehensive understanding of the growth changes in the muscle architecture of MG. Increased knowledge of growth changes in muscle architecture will provide insights into the development of human movements and sports performance during the growth period. Based on previous findings mentioned above, we aimed to test the following hypothesis: muscle thickness, pennation angle and fascicle length will be correlated with the chronological age.

## Methods

### Participants

One hundred forty-six Japanese boys voluntarily participated in this study (age: 6.2–17.9 years, stature: 1.164–1.843 m, body mass: 19.8–78.9 kg). The participants were consisted of non-athletes (*n* = 20) and baseball (*n* = 46), soccer (*n* = 26), track and field (*n* = 18), basketball (*n* = 13), volleyball (*n* = 11), wushu (*n* = 7), and judo (*n* = 5) athletes. The years from the age at PHV were estimated based on, stature, body mass, leg length, and sitting height (calculated by subtracting stature from leg length: Malina et al. 2004 [8]) using the maturity offset method [9]; this was used as the maturity index. All the participants were healthy with no disability and/or disorder in their lower extremities. Prior to the experiments, the purpose of this study and the possible risks associated with the measurements were explained to the participants and their parents, and written informed consent was obtained from the participants and their parents.

### Measurements and analyses of the muscle thickness, pennation angle, and fascicle length of MG

**Fig. 1:**
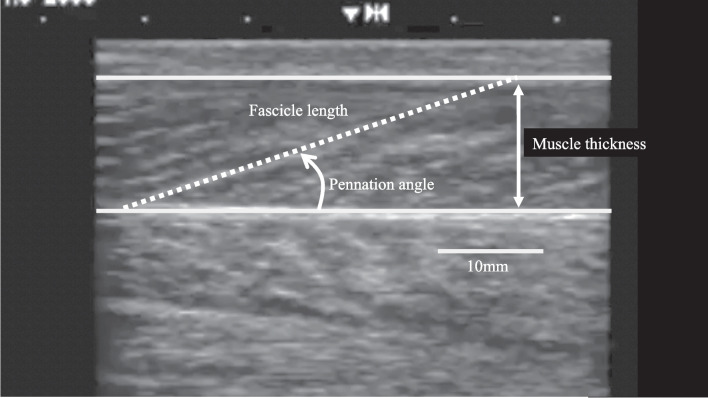
Ultrasound image for measuring the muscle thickness, pennnation angle, and fascicle length of the medial gastrocnemius

The muscle thickness, pennation angle, and fascicle length of MG were measured using Brightness-mode (B-mode) ultrasonography (HS-2000; HONDA ELECTRONICS, Japan) with an electronic linear array probe (HLS-475, 7.5 MHz wave frequency, 50 mm widths; HONDA ELECTRONICS, Japan). An ultrasound image (Fig. 1) was recorded on a secure digital memory card through an analog-to-digital recorder (GV-VCBOX; I-O DATA, Japan). During the measurements, the participants were asked to lay prone on a bed with their legs fully extended and their ankles off the edge of the bed. Each participant was instructed to relax and not contract their muscles during the measurements. Scans were taken from the right leg at 30% of the lower-leg length from the popliteal crease to the malleolus lateralis. The muscle thickness of MG was defined as the perpendicular distance between the deep and superficial aponeuroses. The fascicle length of MG was determined as the length of the fascicular path between the intersections of the fascicle and its deep and superficial aponeuroses. The pennation angle of MG was defined as the angle between the fascicle and the deep aponeurosis. All parameters were analyzed using software (ImageJ; National Institutes of Health, USA). The repeatability of muscle thickness, pennation angle, and fascicle length measurements was tested on two separate days in a preliminary study with five children aged 9.4 − 14.5 years. No significant differences were observed between the test and retest muscle thickness, pennation angle, and fascicle length values. The coefficient of variation was 3.5 ± 3.0% for muscle thickness and 5.0 ± 4.1% and 2.6 ± 2.3% for the pennation angle and fascicle length, respectively.

### Statistics

To exclude the effects of body size, the relative muscle thickness was calculated by dividing the muscle thickness by the body weight^1/3^. A simple regression analysis was used to calculate the Pearson product-moment correlation coefficients for the relationships between the two variables. In order to compare the present and previous results, a simple regression analysis was also performed for the two variables in two age subgroups (5 − 12 years: [7]; 10 − 19 years: [5]).

## Results

**Fig. 2:**
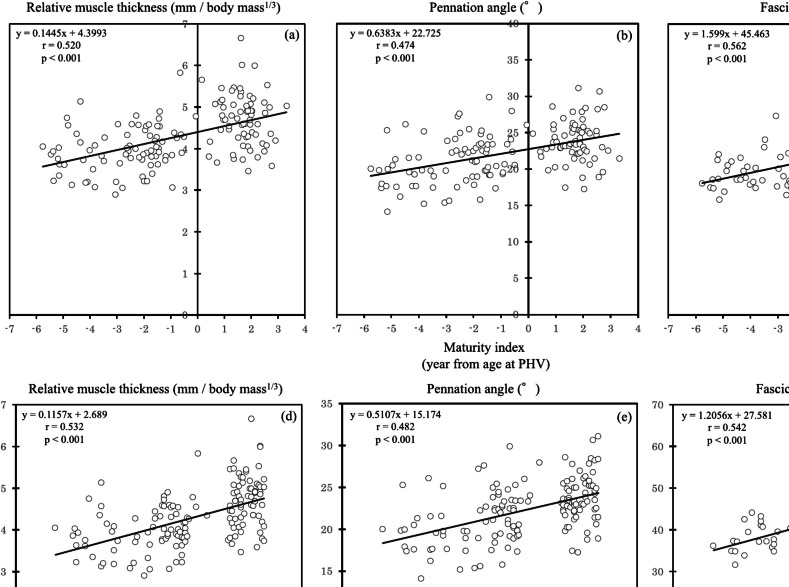
Relationships between the maturity index and chronological age and the relative muscle thickness, pennation angle, and fascicle length of the medial gastrocnemius (maturity index: muscle thickness (**a**), pennation angle (**b**), fascicle length (**c**); chronological age: muscle thickness (**d**), pennation angle (**e**), fascicle length (**f**)). PHV: peak height velocity

The relative muscle thickness, pennation angle, and fascicle length were significantly correlated with the maturity index (muscle thickness: *r* = 0.520, *p* < 0.001; pennation angle: *r* = 0.474, *p* < 0.001; fascicle length: *r* = 0.562, *p* < 0.001) and chronological age (muscle thickness: *r* = 0.532, *p* < 0.001; pennation angle: *r* = 0.482, *p* < 0.001; fascicle length: *r* = 0.542, *p* < 0.001) (Fig. 2).

**Fig. 3:**
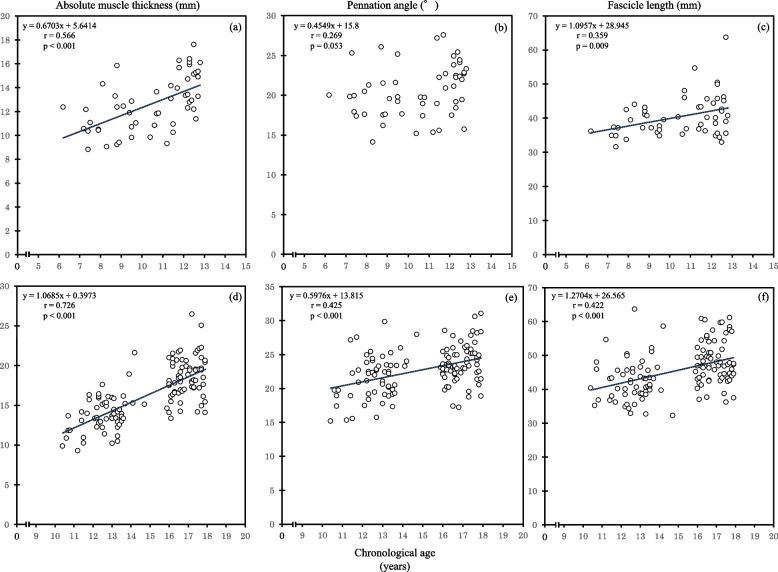
Relationships between the chronological age and absolute muscle thickness, the pennation angle and fascicle length of the medial gastrocnemius in two subgroups (5 − 12 years group: muscle thickness (**a**), pennation angle (**b**), fascicle length (**c**); 10 − 19 years group: muscle thickness (**d**), pennation angle (**e**), fascicle length (**f**))

In subgroups, chronological age was significantly correlated with the absolute muscle thickness (5 − 12 years: *r* = 0.566, *p* < 0.001; 10 − 19 years: *r* = 0.726, *p* < 0.001), pennation angle (10 − 19 years: *r* = 0.425, *p* < 0.001), and fascicle length (5 − 12 years: *r* = 0.359, *p* = 0.009; 10 − 19 years: *r* = 0.422, *p* < 0.001) except for the pennation angle in the 5 − 12 years subgroup (*r* = 0.269, *p* = 0.053) (Fig. 3).

## Discussion

The present results showed that the relative muscle thickness, pennation angle, and fascicle length of MG were positively correlated with the maturity index and chorological age. These results support the present hypothesis and suggest that muscle hypertrophy and the elongation of fascicle length occur during the growth period. Our results also showed that chronological age was correlated with absolute muscle thickness, pennation angle, and fascicle length in both subgroups, except for pennation angle in the 5 − 12 years subgroup. For the 5 − 12 years subgroup, the present results are consistent with a previous finding that the chronological age was correlated with absolute muscle thickness and fascicle length, except for the pennation angle [7]. On the other hand, in the 12 − 19 years subgroup, current results are partially inconsistent with the previous results that the fascicle length was not correlated with the chronological age [5]. The discrepancy between the present and previous results may be attributed to the sample size. The current results, based on a larger sample size, indicate that the fascicle length of MG increases with growth.

Our results showed that the pennation angle was correlated with chronological age in 12 − 19 years subgroup but no such trend was observed in the 5 − 12 years subgroup. These results suggest that the growth changes in pennation angle of MG differ between pre-adolescence and adolescence. The reason for the increasing pennation angle is that a large pennation angle allows more contractile materials to be attached to the aponeurosis area [10]. Meanwhile, because the aponeurosis elongates with growth [5, 7], more contractile materials can be attached to the aponeurosis without a large increase in the pennation angle for the 5 − 12 years subgroup. On the other hand, because muscle size increases rapidly during adolescence [11], it may be assumed that the growth of aponeurosis length may not be sufficient for increased contractile materials to be attached for the 12 − 19 years subgroup. Therefore, the pennation angle may have to increase during adolescence.

This study has some limitations. First, this was a cross-sectional study, and it is possible that our results reflect differences in the underlying characteristics of the participants rather than the growth changes. Therefore, a longitudinal study is needed to confirm the current findings. Second, it is unknown whether the present findings can be applied to the muscles other than MG. Further studies should be performed on muscles other than MG to confirm the present findings.

## Conclusions

This study investigated the correlation between the muscle thickness, pennation angle, fascicle length of MG, and the maturity index and chronological age. The results indicated that the muscle thickness, pennation angle, and fascicle length of MG increased with growth. In addition, the pennation angle was correlated with chronological age in the 12 − 19 years subgroup but no such trend was observed in the 5 − 12 years subgroup, indicating that the growth-related changes in the pennation angle of MG differ between pre-adolescence and adolescence.

## Data Availability

The datasets during and/or analyzed during the current study available from the corresponding author on reasonable request.

## Abbreviations

MG: Medial gastrocnemius
PHV: Peak height velocity
